# IMPACT OF A DRIVING DECISION AID ON DECISIONAL CONFLICT AMONG OLDER ADULT DRIVERS AND THEIR STUDY PARTNERS

**DOI:** 10.1093/geroni/igac059.3057

**Published:** 2022-12-20

**Authors:** Nicole Fowler, Rachel Johnson, Ryan Peterson, Matthew Schroeder, Carolyn DiGuiseppi, Duke Han, Linda Hill, Marian Betz

**Affiliations:** Indiana University School of Medicine, IU Center for Aging Research, Indianapolis, Indiana, United States; Colorado School of Public Health, Aurora, Colorado, United States; University of Colorado, Aurora, Colorado, United States; Regenstrief Institute, Inc., Indianapolis, Indiana, United States; University of Colorado Anschutz Medical Campus, Aurora, Colorado, United States; University of Southern California, Alhambra, California, United States; University of California San Diego, La Jolla, California, United States; University of Colorado School of Medicine, Aurora, Colorado, United States

## Abstract

Forty-four million US licensed drivers are ≥65 years old and at higher crash risk. Decision-making about stopping or continuing driving is difficult and often involves family and friends. This study examines if decision conflict about changing driving habits is associated between older adult drivers and their study partners (SPs) (i.e., family member or friend). Data were from a multi-site trial assessing a driving decision aid. Decision conflict about stopping or continuing driving for drivers and their SPs were measured with the Decision Conflict Scale (DCS). Dyadic associations between drivers’ and SPs’ DCS scores pre- and post-decision aid implementation were analyzed using an actor-partner interdependence model. Among 228 driver-SP dyads, driver mean (SD) age was 77.1 (5.1) years; 50.0% female; 98.7% non-Hispanic; 94.7% white; and 97.8% urban-dwelling. SPs mean age was 66.1 years (13.9); 65.8% female; 95.6% non-Hispanic; 92.1% white; and commonly the driver’s spouse (54.6%) or adult child (21.1%). Most drivers (71.7%) and SPs (63.3%) had baseline DCS scores < 25 (drivers mean 18.5 (SD 12.3); SPs 20.5 (16.8)), suggesting low decision conflict. DCS was correlated within dyads at baseline (r=0.18, p < 0.01), and baseline DCS was associated with post-decision aid DCS (p < 0.001 for SPs [β=0.73] and drivers [β=0.73]). While SPs’ baseline DCS was not associated with drivers’ post-decision aid DCS, drivers’ baseline DCS and SPs’ post-decision DCS were (β=0.10; p=0.036). Higher decision conflict about driving felt by older drivers is frequently shared by their SPs, in whom decision conflict may persist even after a driving decision aid intervention.

